# Modern Methods of Vaccination

**Published:** 1902-02-15

**Authors:** 


### MODERN METHODS OF VACCINATION.

The last three meetings of the Royal Medical and Chirurgical Society have been occupied in discussing modern methods of vaccination. The discussion was opened by Dr. Monckton Copeman, who gave a full and careful account of the history and the present position of vaccination as a prophylaxis against small pox, and more especially described the modern method by glycerinated calf lymph which has now been adopted officially and in the elaboration of which Dr. Copeman has himself played so large a part. As a fact very little was added by the other speakers beyond what was stated in his very lucid address. Referring to the degree of success which had been attained by the public vaccinators using this lymph, he said that during the first year of operations nearly 500,000 tubes of this lymph were sent out from the Government laboratories and that, notwithstanding the novelty of the method, the returns showed that a case-success of 93 per cent. and an insertion-success of 83 per cent. had been attained. In a recent lymph series which he had officially investigated he had found that the case-success and the lymph-success were 98 per cent. and 93 per cent. respectively, while it was now no unusual thing at the Government laboratories to receive reports showing complete success both as to cases and insertions. Speaking of the details of the operation he said that of the various preparations used for insuring cleanliness of the skin a warm solution of boric acid is in all probability the most generally useful, stronger antiseptics, unless thoroughly removed by the subsequent use of sterilised water or alcohol, being liable to exert a somewhat prejudicial effect upon the lymph. Various substances are used by different operators for the protection of the vaccinated areas during the first few days, but it is essential during the second week of the process that some dressing of an absorbent nature should be employed, as during this period oozing from the vesicles occasionally supervenes. At the Government station at Lamb's Conduit Street, a dressing composed of a couple of layers of boric lint kept in position by means of pieces of india-rubber strapping, which do not entirely encircle the arm, is applied at the time of the vaccination, and this is replaced by another exactly similar dressing when a week later the case returns for inspection. But whatever be the nature of the dressing the free use beneath it of a dusting powder of boric acid has been found most beneficial in preventing any undue amount of inflammatory reaction. As to instruments, possibly the best and certainly the simplest is the ordinary triangular-headed surgical needle. The manner of operating which affords the most generally successful results consists in blowing out the lymph from the tube on to the surface of the skin at different points corresponding to the situations of the vesicles which it is desired to obtain. The skin, put slightly on the stretch, is then scarified through each droplet of lymph with the needle, first in one direction and then in another more or less at right angles to the first, the drawing of blood being avoided as far as possible. Many operators, Dr. Copeman says, reverse the procedure somewhat, first making their scarification, and then rubbing in the lymph, but certain it is that the results thus obtained are by no means comparable with those following on the method of scarifying through the beads of lymph previously dropped upon the skin. On completing the operation it is desirable to avoid too great haste in applying the protective dressing, especially if this be of the nature of an absorbent pad, or if it be impregnated with some powerful germicide such as sal alembroth wool. A point of considerable importance is that the permanent scarring of the skin produced by modern methods may be "astonishingly slight," so that the whole of our ideas in regard to the importance of the vaccination marks may in the uture have to be reviewed. In the discussion Dr. T. D. Acland expressed the hope, which we think will be echoed by most medical men, that the Government would take steps to establish a laboratory on a far larger scale than at present, so that all practitioners in the kingdom might be able to obtain lymph produced under conditions unfettered by the economies necessary in establishments run solely for trade purposes, and he gave notice of a resolution to the effect that Government should supply all practitioners with glycerinated lymph, that all imported lymph should be examined, and that all manufactories of lymph should be inspected. Dr. Sidney Coupland thought that the profession had perhaps not considered sufficiently the slighter injury which followed vaccination. In the majority of cases in which bad results followed it was not the lymph which was at fault, but some accidental complication, and he quite agreed with what Dr. Acland had said about the desirability of Government having the full control of all vaccine used in this country.

